# Nerve Excitability in Asymptomatic Carriers and Amyotrophic Lateral Sclerosis Patients With C9orf72

**DOI:** 10.1002/acn3.70187

**Published:** 2025-09-09

**Authors:** Diederik J. L. Stikvoort García, H. Stephan Goedee, Leonard H. van den Berg, Boudewijn T. H. M. Sleutjes

**Affiliations:** ^1^ Department of Neurology, Brain Centre Utrecht University Medical Centre Utrecht Utrecht the Netherlands

**Keywords:** amyotrophic lateral sclerosis, C9orf72, nerve excitability

## Abstract

**Objective:**

We investigated the effects of C9orf72 mutation carriership on peripheral nerve excitability in asymptomatic individuals from families with a history of C9orf72 amyotrophic lateral sclerosis (ALS) and patients.

**Methods:**

We included 47 asymptomatic individuals from families with a history of C9orf72 ALS, of whom 23 were carriers (C9^+^) and 24 were noncarriers (C9^−^). In addition, 11 C9^+^ and 110 C9^−^ ALS patients and 50 healthy controls participated. Nerve excitability tests were conducted on the median nerve. We obtained standard excitability measurements as well as composites of these measurements that reflect various passive and active membrane properties. Data of C9^+^ and C9^−^ asymptomatic individuals were compared, followed by a kinship‐adjusted comparison in asymptomatic individuals from the same families. We then compared C9^+^ to C9^−^ ALS patients.

**Results:**

In the subset of asymptomatic individuals from the same families, C9^+^ individuals had lower values than C9^−^ individuals on one of the composite excitability measurements (*t* = −2.15, *p =* 0.034), corresponding to a hypoexcitable profile consistent with smaller Na^+^‐window currents. C9^+^ ALS patients had a hyperexcitable profile with larger refractoriness at 2 ms and relative refractory periods than C9^−^ ALS patients (*t* = 4.58, *p* < 0.001; *t* = 3.43, *p* = 0.002, respectively), which is in line with slower recovery of the Na^+^‐channels from inactivation.

**Interpretation:**

Asymptomatic individuals and ALS patients carrying the C9orf72 mutation exhibit a unique electrophysiological phenotype, implicating altered Na^+^‐channel characteristics compared to asymptomatic noncarriers and sporadic ALS patients. Monitoring hypoexcitable to hyperexcitable profile transitions in individuals carrying the C9orf72 mutation may be valuable as an early indicator of phenoconversion.

## 1 Introduction

Amyotrophic lateral sclerosis (ALS) is a devastating neurodegenerative disorder affecting both upper and lower motor neurons, causing weakness, respiratory failure, and eventually death [1]. Genetic and environmental factors are believed to play a major role in the pathogenesis of ALS [2]. The most prevalent ALS‐linked genetic mutation is the hexanucleotide repeat expansion (GGGGCC) in the C9orf72 gene, accounting for approximately 40% of familial ALS cases and 6% of sporadic cases in Europe and North America [3]. However, the penetrance of the mutation is incomplete [4, 5], introducing substantial uncertainty about an individual's risk of developing ALS. Ultimately, facilitating timely intervention in the presymptomatic phase, before the onset of disabling motor neuron loss, will require biomarkers that can identify individuals with a high risk of developing ALS and that can detect early signs of disease onset [6].

Multiple modalities have already been implemented to study asymptomatic C9orf72 carriers. Imaging studies have found differences in the structure of various cerebral regions between asymptomatic C9orf72 mutation carriers and noncarriers [7, 8, 9, 10, 11], which have helped to identify high‐risk individuals [12]. Increases in neurofilament light chain (NfL) concentrations have also been reported in asymptomatic mutation carriers, including C9orf72 [6]. Although these promising biomarkers are strong indicators of ongoing neurodegeneration, critical pathophysiological changes in the excitability of motor neurons have been shown to occur well before detectable motor unit loss [13, 14, 15]. Nerve excitability measures have also been found to track target engagement of various ion channel modulating compounds in ALS [16, 17, 18]. Combined, this neurophysiological technique holds potential as a sensitive method for early disease detection and as a tool to support drug discovery. However, it is unclear whether the identified changes in excitability are generalizable to individuals with the C9orf72 mutation, especially during early or presymptomatic stages. Studies of induced pluripotent stem cell (iPSC) derived motor neurons from C9orf72 ALS patients and healthy controls have shown differences in their excitability despite remaining viable [19, 20]. However, supporting studies on the effect of C9orf72 carriership on in vivo peripheral nerve excitability properties are limited [21].

Therefore, the primary aim of this study was to compare peripheral nerve excitability between asymptomatic C9orf72 carriers and noncarriers, with an emphasis on individuals from the same families. In addition, we aimed to compare measurements from C9orf72 ALS patients and sporadic ALS patients to establish whether carriership of the mutation corresponds to a distinct electrophysiological phenotype.

## 2 Materials and Methods

### 2.1 Study Participants

This study was approved by the medical ethics committee of the University Medical Centre Utrecht and performed in accordance with the Declaration of Helsinki. All participants provided written informed consent prior to participation. The final study cohort consisted of: (1) asymptomatic individuals from families with C9orf72 ALS, including carriers (C9^+^) and noncarriers (C9^−^); (2) patients with C9orf72 ALS and sporadic ALS; and (3) healthy (population‐based) controls.

Asymptomatic individuals from families with C9orf72 ALS with no history of peripheral nerve disorders that could potentially influence nerve excitability testing were eligible for participation [17]. Eligibility was not contingent on the participants knowing whether they were carriers of the C9orf72 mutation. The examiner (DS) remained blinded to their respective mutation status at the time of neurophysiological assessment. Between August 2021 and January 2024, we included 47 asymptomatic individuals, all of whom were screened for C9orf72 repeat expansion with ≥ 30 GGGGCC repeats to establish who were carriers (C9^+^) or noncarriers (C9^−^). As part of research in our outpatient clinic, individuals from families with a history of ALS routinely undergo a standardized neurological examination [22]. Whenever available, at most 1 year before participation, or at any point after participation, these data were used to ensure that asymptomatic C9^+^ individuals were indeed asymptomatic. We defined asymptomatic by the absence of signs of upper or lower motor neuron disease and cognitive or behavioral changes.

Additionally, we included 121 ALS patients who fulfilled the Gold‐Coast Criteria [23], who had undergone genetic screening for the C9orf72 repeat expansion and who did not have another known ALS‐associated mutation (e.g., FUS, SOD1, ATXN2). These patients were part of a large prospective study that was performed during their first diagnostic workup at our outpatient clinic, described in detail elsewhere [13]. We used the following exclusion criteria: cognitive dysfunction hampering compliance with the study (e.g., severe frontotemporal dementia); coincidental active neuropathies; use of medication that could affect nerve excitability measures, including riluzole; inability to tolerate electrical nerve stimulation; or absence of motor responses in both abductor pollicis brevis muscles. The examiner (DS) was blinded to patients' diagnoses or the presence of genetic mutations at the time of recording. Clinical staging of the patients with ALS was performed using the revised ALS functional rating scale (ALSFRS‐R, scores range from 0 to 48), the ALSFRS‐R fine motor function subdomain score (FMF, scores range from 0 to 12) and the results from the neurological examination.

Reference nerve excitability recordings were obtained from 50 healthy controls (27 males; mean age, SD = 62, 10) recruited from a prospective population‐based register in The Netherlands [24] and a previous study [13]. These controls were age‐ and gender‐matched to our cohort of ALS patients.

### 2.2 Neurophysiological Protocol

Recordings were performed and processed using the QTRAC software (QTRAC, Institute of Neurology, Queen Square, London, UK) for control and acquisition of the signals (BNC‐6431, National Instruments, Austin, TX). We recorded compound muscle action potentials (CMAP) from the abductor pollicis brevis (APB) using an EMG apparatus (D440, Digitimer, Welwyn Garden City, UK) coupled to a noise eliminator (Humbug, Digitimer, Welwyn Garden City, UK). Recordings were performed on the right hand when possible; the left arm was chosen for recordings if the right APB was paralytic or if severe motor conduction slowing was present that could indicate the presence of carpal tunnel syndrome. The median nerve was stimulated at the wrist level using an isolated bipolar constant stimulator (DS5, Digitimer, Welwyn Garden City, UK). Prior to recording, we warmed participants' arms using a 37°C water blanket (Norm‐O‐Temp & Maxi‐Therm Lite; Cincinnati Sub‐Zero LLC, Cincinnati, Ohio) for 30 min. This blanket was kept in place during all subsequent recordings to minimize temperature‐induced variability [25].

We performed CMAP‐scans—detailed stimulus–response curves—in which all motor units innervating a muscle are gradually recruited [26, 27]. A progressively stepwise pattern in the CMAP‐scan corresponds to progressive motor unit loss. We used the well‐established MScanFit program [28, 29, 30] in order to translate these scans into motor unit number estimates (MUNE). These measures were used to determine whether the nerve excitability tests in the asymptomatic family members were performed in muscles without objectively detectable axon loss. As CMAP‐scan based MUNE can detect subclinical loss of motor units [31], we considered this measurement the most relevant staging variable for the asymptomatic C9^+^ and C9^−^ individuals.

**FIGURE 1:**
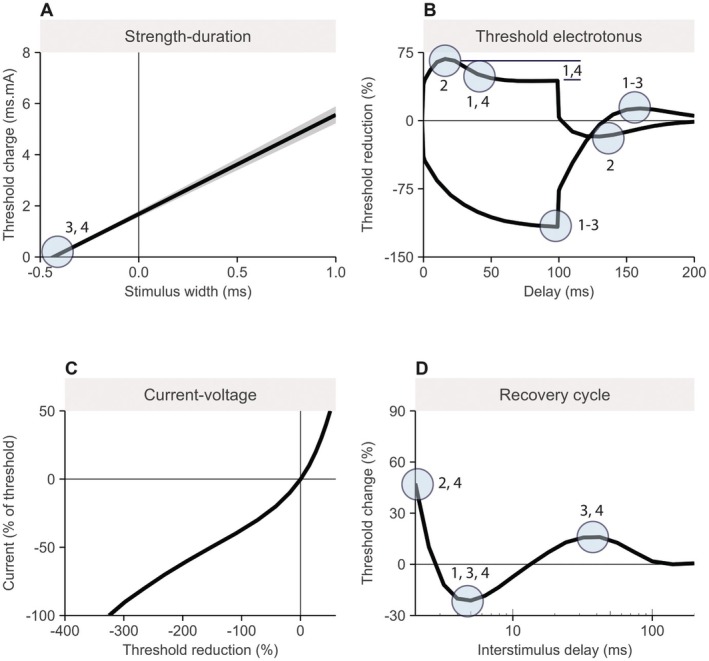
Example of the nerve excitability tests and the components of the composite excitability measurements. Panels represent (A) the strength‐duration test, (B) threshold electrotonus test, (C) current–voltage test, and (D) the recovery cycle. Encircled regions in the tests represent the standard excitability measurements that are used to calculate the biophysically informed composite excitability measurements. The numbers indicate to which composite measurements each point corresponds. For example, superexcitability—the lowest threshold change in panel (D) – contributes to composites 1, 3, and 4.

Standardized nerve excitability tests were performed in line with previous work from our institute [32, 33, 34] and international consensus guidelines [35]. Briefly, nerve excitability tests revolve around quantifying threshold changes to maintain a target response of 40% of maximum CMAP amplitude. We performed the following well‐established tests from the TROND protocol [36]:

- the strength‐duration test (relation between stimulus‐charge and stimulus‐durations from 0.2 to 1.0 ms; Figure 1A)
- the threshold electrotonus test (threshold changes to 20% and 40% depolarizing and hyperpolarizing conditioning currents up to 100 ms; Figure 1B)
- the current–voltage test (threshold changes to 200 ms conditioning currents between 50% depolarizing to 100% hyperpolarizing; Figure 1C)
- the recovery cycle test (threshold changes after supramaximal stimuli at decreasing interstimulus intervals from 200 ms to 2 ms; Figure 1D).

From these tests, we derived a standard set of excitability measurements, including the strength‐duration time‐constant (SDTC), TEd_peak_, TEd_40‐60ms_, TEd_90‐100ms_ and TEh_90‐100ms_ (threshold changes during the threshold electrotonus after 40% depolarizing or hyperpolarizing currents at the peak or given intervals), S2‐accommodation (the difference between TEd_peak_ and TEd_90‐100ms_), TEd_undershoot_ and TEh_overshoot_ (minimum and maximum threshold change after the 100 ms conditioning window at 40% depolarizing and hyperpolarizing currents), the resting, minimal, and hyperpolarizing slopes of the current–voltage test, refractoriness at 2 ms, relative refractory period (RRP), super‐ and subexcitability (maximum and minimum threshold of the recovery cycle after the refractory period).

### 2.3 Biophysically Informed Composite Excitability Measurements

Various mechanisms play a crucial role in the propagation of action potentials and the maintenance of axonal excitability in human myelinated motor axons [37, 38, 39]. The initiation of the action potential is primarily regulated by properties of transient Na^+^‐channels clustered at the node of Ranvier [37]. A small portion of these Na^+^‐channels is opened at lower resting membrane potentials and hardly inactivate (“persistent” Na^+^‐channels; 1%–2%), resulting in persistent inward currents that may increase axonal excitability. After depolarization, the recovery rate of the transient Na^+^‐channels from inactivation drives the refractory periods [38]. Other ion‐channels and pumps, such as fast and slow K^+^‐channels, HCN‐channels, and the Na^+^/K^+^‐pump also aid in maintaining axonal excitability. Combined, the finely tuned activity of these ion‐channels ensures that human myelinated motor axons maintain efficient and reliable action potential propagation. For example, HCN‐channels open during long‐lasting hyperpolarization to allow inward rectifying currents (HCN channels), thereby driving the membrane potential to less negative values and preventing severe hyperpolarization [39]. Due to the underlying (patho)physiology properties, subtle differences or alternations of one or more of these mechanisms may affect multiple excitability parameters, yielding a characteristic pattern of excitability changes.

We recently showed that recordings of peripheral nerve excitability in ALS patients can be separated into four independent patterns of changes—representations of how each recorded datapoint changes with respect to the others, rather than individually—that were driven by several of the aforementioned biophysical mechanisms [40]. The most likely mechanisms corresponding to these patterns, in order of percentage variance contributed, were: (1) membrane polarization (modulated by Na^+^/K^+^‐pump currents, 36%), with lower values indicating membrane depolarization; (2) gating kinetics of slow K^+^‐channels (18%), with larger values corresponding to less steep activation slopes; (3) gating kinetics and permeability of Na^+^‐channels, with larger values corresponding to larger total Na^+^‐window currents (10%); and (4) the refractoriness of the nerve (9%). These patterns can be closely approximated by combining subsets of the recorded standard excitability measurements into four composite excitability measurements (Figure 1). The equations to calculate each composite measurement are:

$$\begin{array}{cc} \text{Composite}\;1=\; & -3.92\times {\mathrm{TEh}}_{90-100\mathrm{ms}}+2.57*{\mathrm{TEd}}_{40-60\mathrm{ms}}+1.91\\ & \times \text{Superexcitability}-0.26\times {\mathrm{TEh}}_{\text{overshoot}}-0.07\times S2 \end{array}$$

$$\begin{array}{cc} \text{Composite}\;2=\; & 3.00\times {\mathrm{TEd}}_{\text{undershoot}}+2.06\times {\mathrm{TEh}}_{90-100\mathrm{ms}}-1.15\\ & \times {\mathrm{TEh}}_{\text{overshoot}}+1.022\times {\mathrm{TEd}}_{\text{peak}}-0.20\times \text{Refractoriness} \end{array}$$

$$\begin{array}{cc} \text{Composite}\;3=\; & 2.00\times {\mathrm{TEh}}_{90-100\mathrm{ms}}+1.80\times \text{Superexcitability}+1.71\\ & \times {\mathrm{TEh}}_{\text{overshoot}}+0.79\times \text{SDTC}-0.63\times \;\text{Subexcitability} \end{array}$$

$$\begin{array}{cc} \text{Composite}\;4=\; & 1.77\times \text{Refractoriness}-1.53\times \text{SDTC}+0.88\\ & \times {\mathrm{TEd}}_{40-60\mathrm{ms}}+0.43\times S2+0.37\times \text{Superexcitability} \end{array}$$

The measurements in the equations were first standardized with respect to controls, such that each unit increase corresponds to one standard deviation, for example, [*x*_*i*_ – *μ*(*x*_controls_)]/*σ*(*x*_controls_). Compared to the individual standard excitability measurements, the primary advantage of these biophysically informative composite measurements is that they generate insights into the mechanisms that regulate axonal excitability at the individual level. Conventional statistical methods can then be applied to study these mechanisms directly from recordings. Therefore, we used these composite excitability measurements as the main outcome for studying the effects of C9orf72 mutation carriership. The standard excitability measurements were assessed as secondary outcomes to capture potential effects that may not be adequately described by the composite measurements.

### 2.4 Statistical Analysis

Differences in group characteristics between the asymptomatic C9^+^ and C9^−^ individuals, as well as between C9^+^ and C9^−^ ALS patients, were examined using Mann–Whitney U tests for continuous variables, Fisher's exact tests for categorical variables, and cumulative link models for ordinal variables, such as reflex severity or medical research council scores. Standard measurements obtained from the nerve excitability tests, as well as the novel composite excitability measurements, were compared between groups in two analyses: (1) we cross‐sectionally compared all asymptomatic C9^+^ individuals to all C9^−^ individuals and to healthy controls, followed by a kinship adjusted comparison between only asymptomatic C9^+^ and C9^−^ individuals from the same families; and (2) a comparison of C9^+^ ALS patients to C9^−^ ALS patients and to healthy controls. Except for the kinship adjusted comparison, all statistical analyses were performed using generalized linear models. Age and gender were incorporated as covariates in the models as these may influence nerve excitability measurements [41, 42].

For analysis of asymptomatic individuals from the same families, we constructed a kinship matrix based on their pedigrees to be used as the covariance matrix of random effects in a linear mixed effects model. With this approach, differences between individuals with high kinship coefficients (e.g., siblings) are weighted stronger than those between individuals with lower kinship coefficients (e.g., cousins). Given that, with the exception of the C9orf72 mutation, individuals with higher kinship coefficients are more genetically similar, this technique is considered to enhance power to detect a potential gene‐driven phenotype [10, 11]. As these analyses were performed in a smaller sample, age and gender were only added as additional covariates to the models when these reduced the model AIC to reduce the risk of overfitting. *P* values from all group comparisons were adjusted for multiple testing with Tukey's honest significant difference test.

All statistical analyses were performed using R (R Core Team, 2020, R Foundation for statistical computing, Vienna, Austria, http://cran.r‐project.org). *P* values < 0.05 were considered significant.

## 3 Results

### 3.1 Demographics

We included a total of 23 (49%) asymptomatic C9^+^ and 24 (51%) C9^−^ individuals. Of the ALS patients, 11 (9%) were C9^+^ and 110 (91%) C9^−^. None of the patients were related to any of the included asymptomatic individuals. Demographic and clinical characteristics of the study population are summarized in Table 1. There were no differences in any of the demographic variables between the groups of asymptomatic individuals (C9^+^ vs. C9^−^) or the ALS patients (C9^+^ vs. C9^−^). The most relevant muscle staging variable, MUNE, did not differ between the asymptomatic groups (*W* = 260, *p* = 0.733). Neurological examination results were available for 22/23 (96%) C9^+^ and 23/24 (96%) C9^−^ asymptomatic family members, none of whom exhibited signs of lower motor neuron dysfunction or upper motor neuron dysfunction (Supporting Information). Non‐subthreshold conditioning artifacts were observed in one C9^+^ and one C9^−^ individual, and their corresponding measures were omitted from analyses.

**TABLE 1:** Characteristics of the study population.

Characteristic	Asymptomatic individuals		ALS	
	C9^+^	C9^−^	C9^+^	C9^−^
Demographics				
Participants (n)	23	24	11	110
Age, years	49 (10)	53 (13)	60 (7)	63 (9)
Sex (m/f)	8/15	13/11	4/7	70/40
Disease duration (months)	—	—	13 (7)	14 (16)
Staging				
MUNE	94 (31)	91 (21)	54 (33)	51 (32)
MRC (5/4/3/2)	23/0/0/0	24/0/0/0	7/4/0/0	65/38/6/1
ALSFRS‐R	—	—	42 (4)	41 (4)
FMF	—	—	11 (9, 12)	10 (9, 12)
dF	—	—	−0.6 (0.4)	−0.8 (0.9)

*Note:* Data are presented as count (*n*) or mean (standard deviation).

Abbreviations: ALSFRS‐*R*, revised ALS functional rating scale; C9^+^, C9^−^, positive or negative for the C9orf72 repeat expansion; dF, ALSFRS‐R slope since onset; FMF, fine motor function; MRC, medical research council scores (range = 0–5); MUNE, motor unit number estimates (thenar muscles).

### 3.2 Excitability Properties in Asymptomatic C9orf72 Carriers

**FIGURE 2:**
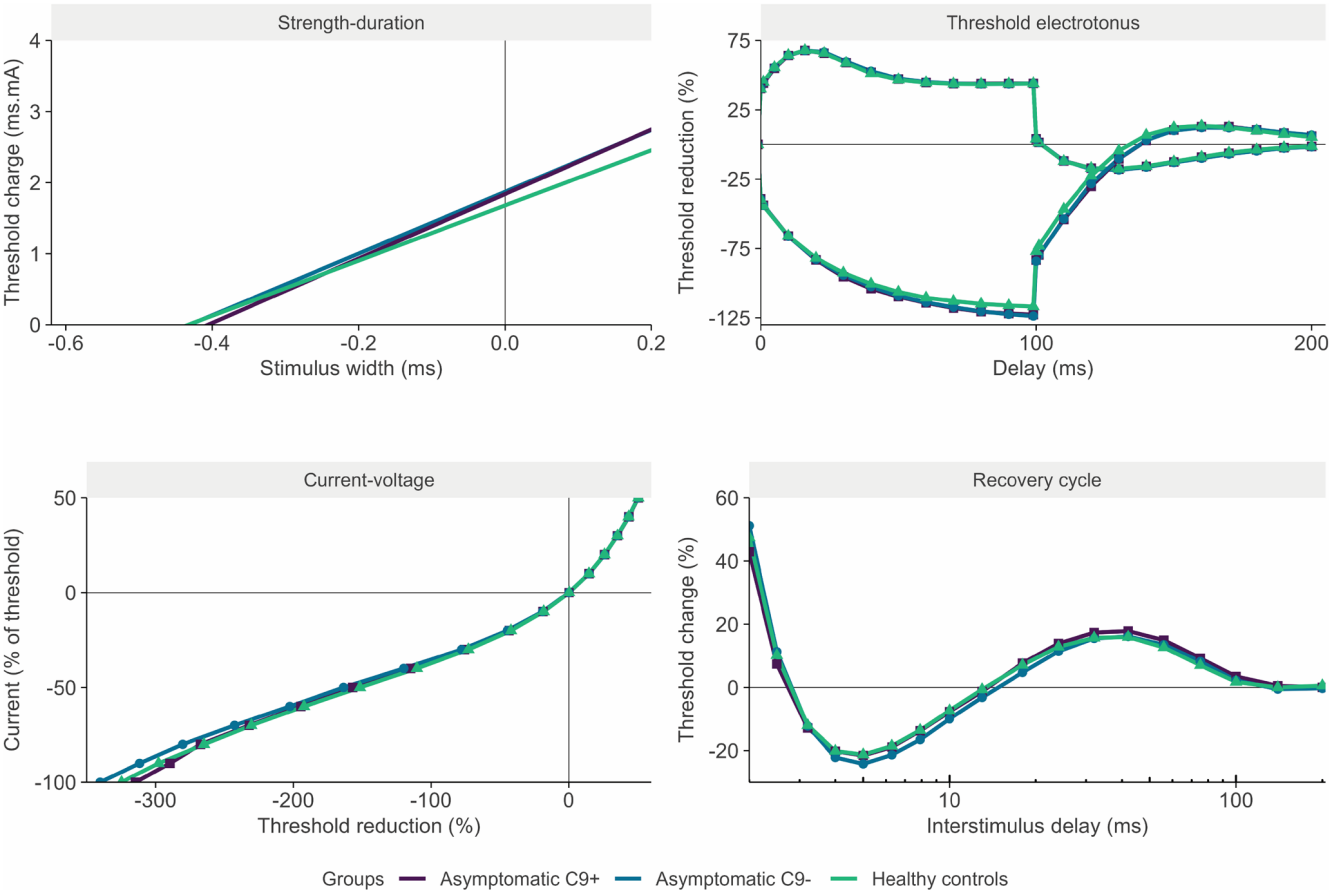
Comparison of nerve excitability recordings and corresponding measurements from the asymptomatic carriers, noncarriers, and healthy controls. Panels represent the average results from the four standard excitability tests from the asymptomatic individuals with and without a C9orf72 mutation (C9+ and C9^–^), as well as healthy, population‐based controls. No significant differences were found between the three groups.

**FIGURE 3:**
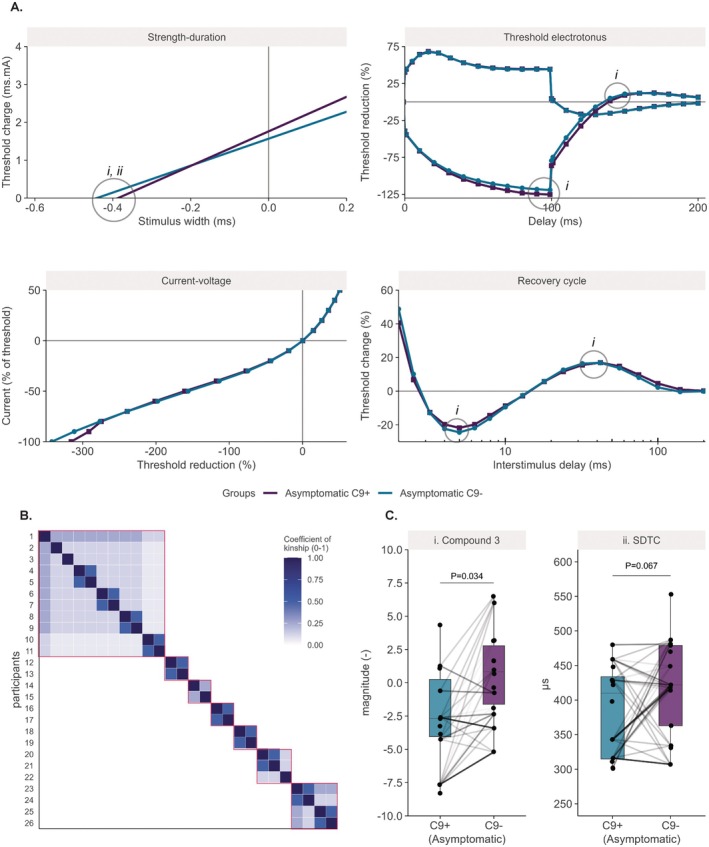
Comparison of nerve excitability recordings and corresponding measurements from related asymptomatic carriers and noncarriers. Panels in (A) represent the average results from the four standard excitability tests from asymptomatic individuals with and without a C9orf72 mutation (C9+ and C9^–^). (B) Depicts the constructed kinship matrix for the subset of related asymptomatic individuals as a heatmap, with each red square encompassing one distinct pedigree. Panels in (C) depict composite excitability measurement 3 and the strength‐duration time‐constant (SDTC), with their origins detailed in panel A; the lines between the C9+ and C9^–^ groups illustrate the inter‐individual relations, with darker lines corresponding to a higher kinship coefficient. For legibility, only kinships between the two groups are displayed, but not those within the groups. *P* values were obtained from linear mixed effects models as described in the statistics section.

The resulting electrophysiological outcomes are summarized in Table 2. Overall, no significant differences were identified between asymptomatic C9^+^ and C9^−^ individuals without accounting for kinship between related individuals (Figure 2). Similarly, no measurement was found to significantly differ between controls and asymptomatic C9^+^ individuals. We then zoomed in on the excitability measurements of asymptomatic C9^+^ and C9^−^ carriers from the same families. This subset included a total of 26 (55%) individuals, of whom 12 (46%) were C9^+^. Figure 3A illustrates the averaged recordings of these related asymptomatic individuals grouped by mutation status. This subset of participants consisted of relatives from seven different pedigrees (Figure 3B). Age and gender were comparable between the C9^+^ and C9^−^ groups in this subset (*W* = 108.5, *p* = 0.217; odds‐ratio = 0.39, *p* = 0.267), as was MUNE (*W* = 73.5, *p* = 0.607). Again, no significant differences were found between the standard excitability measurements of these two groups, even though we accounted for interfamilial genetic variability using the kinship matrix. Composite excitability measurement three values, however, indicative of nodal Na^+^‐currents, were lower in the asymptomatic C9^+^ individuals (mean ± standard error: C9^+^ = −2.41 ± 1.09, C9^−^ = 0.70 ± 0.90; *t* = −1.93, *p* = 0.034; Figure 3C). The standard excitability measurements that make up this composite did not significantly differ between the groups, including the TEh_90‐100ms_, TEh_Overshoot_, super‐ and subexcitability, and the SDTC. Of these, the SDTC had the largest effect size and thus the largest contribution to composite measurement 3 (mean ± standard error: C9^+^ = 386 ± 19 μs, C9^−^ = 441 ± 25 μs; *t* = −1.65, *p* = 0.067; Figure 3C). Still, the group differences in these measurements uniformly contributed to a reduction in composite 3, indicating that the underlying data accurately captured the expected pattern of excitability changes. As a result, the observed differences in excitability are most consistent with smaller Na^+^‐window currents in the asymptomatic C9^+^ individuals with respect to their asymptomatic C9^−^ relatives.

**TABLE 2:** Excitability measurements of the study population.

Characteristic	Healthy controls	Asymptomatic individuals		ALS	
		C9^+^	C9^−^	C9^+^	C9^−^
Strength‐duration test					
SDTC (μs)	433 (8)	406 (14)	431 (17)	494 (24)	451 (9)
Rheobase (mA)	3.9 (0.2)	4.5 (0.4)	4.3 (0.4)	4.2 (0.4)	4.1 (0.2)
Threshold electrotonus test					
TEd_Peak_ (%)	65.3 (0.5)	65.2 (0.7)	65.7 (0.8)	68.0 (1.7)	67.2 (0.5)^c^
TEd_40‐60ms_ (%)	47.2 (0.5)	47.7 (0.7)	48.1 (0.9)	50.9 (1.6)	51.2 (0.4)^c^
TEd_90‐100ms_ (%)	43.8 (0.5)	43.9 (0.6)	43.7 (0.8)	45.3 (1.0)	47.8 (0.5)^c^
TEh_90‐100ms_ (%)	−116 (2)	−122 (4)	−123 (4)	−121 (5)	−120 (2)
S2‐accommodation (%)	21.5 (0.4)	20.8 (0.7)	21.8 (0.7)	23.1 (1.4)^b^	19.6 (0.4)^c^
TEh_Overshoot_ (%)	13.9 (0.5)	13.6 (1.0)	13.9 (0.9)	16.3 (1.7)	13.5 (0.5)
TEd_Undershoot_ (%)	−17.2 (0.3)	−17.6 (0.9)	−18.0 (0.7)	−19.3 (1.6)	−16.3 (0.4)
Current–voltage test					
Resting slope (−)	0.6 (0.0)	0.6 (0.0)	0.6 (0.0)	0.6 (0.0)	0.6 (0.0)^c^
Hyperpolarizing slope (−)	0.3 (0.0)	0.4 (0.0)	0.3 (0.0)	0.3 (0.0)	0.4 (0.0)
Minimal slope (−)	0.2 (0.0)	0.2 (0.0)	0.2 (0.0)	0.3 (0.0)	0.3 (0.0)
Recovery cycle test					
Refractoriness at 2 ms (%)	47.4 (2.3)	42.9 (3.8)	51.2 (4.3)	78.1 (12.0)^b^, ^c^	42.3 (2.5)
RRP (ms)	2.8 (0.0)	2.7 (0.1)	2.8 (0.1)	2.9 (0.1)^b^	2.6 (0.0)^c^
Superexcitability (%)	−20.1 (0.8)	−20.4 (0.7)	−22.9 (1.2)	−25.6 (2.1)	−26.1 (0.7)^c^
Subexcitability (%)	15.3 (0.6)	17.2 (1.4)	15.8 (1.6)	15.9 (2.5)	14.0 (0.5)
Composite measures of excitability					
Composite 1 (−)	0.0 (0.9)	2.1 (1.4)	3.8 (1.4)	5.4 (2.5)	5.0 (0.8)^c^
Composite 2 (−)	0.0 (0.7)	−0.4 (1.5)	−1.9 (1.1)	−2.4 (2.2)	1.7 (0.8)^c^
Composite 3 (−)	0.0 (0.4)	−1.5 (0.8)^a^	−0.1 (0.8)	3.1 (1.3)	1.9 (0.4)
Composite 4 (−)	0.0 (0.4)	0.5 (0.6)	1.1 (0.8)	2.5 (1.1)	0.0 (0.3)

*Note:* Data are presented as mean (standard error).

Abbreviations: ALS, amyotrophic lateral sclerosis; C9^+^, C9^−^, positive or negative for the C9orf72 repeat expansion.

^a^ Significant differences with respect to asymptomatic C9^−^ (accounting for kinship).

^b^ Significant differences with respect to C9^−^ ALS.

^c^ Significant differences with respect to healthy controls.

### 3.3 Excitability Properties in C9orf72 ALS Patients

**FIGURE 4:**
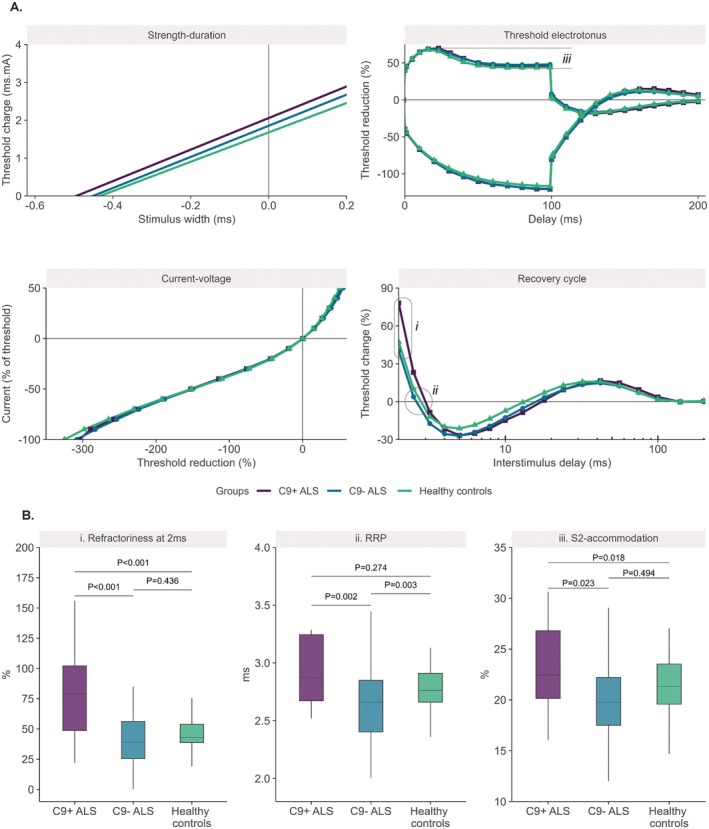
Comparison of nerve excitability recordings and corresponding measurements in ALS patients and healthy controls. Panels in (A) represent the average results from the four standard excitability tests from ALS patients with and without a C9orf72 mutation (C9+ and C9^–^). Panels in (B) depict the refractoriness at 2 ms, the relative refractory period (RRP) and the S2‐accommodation, with their origins detailed in panel A. *P* values were obtained from linear models as described in the statistics section.

The average recordings of C9^+^ ALS patients were largely comparable to the C9^−^ ALS patients, with the most notable difference present in the refractory region of the recovery cycle (Figure 4A). Changes in this region are largely dominated by the recovery of Na^+^‐channels from inactivation. C9^+^ patients exhibited larger refractoriness at 2 ms, RRP, and S2‐accommodation compared to C9^−^ patients (*t =* 4.58, *p* < 0.001; *t* = 3.43, *p* = 0.002; and *t =* 2.66, *p* = 0.023, respectively, Figure 4B), which suggests a hyperexcitable profile due to slower recovery from inactivation that prolongs the influx of Na^+^‐ions into the axoplasm. Although the large refractoriness at 2 ms in C9^+^ ALS patients was a major driver of the mean value of composite excitability measurement 4 in this group, the total contribution of the other standard excitability measurements underlying the composite (SDTC, TEd_40‐60ms_, S2 and superexcitability) was negligible. Consequently, the difference between composite measurement 4 in C9^+^ ALS and C9^−^ ALS patients did not reach significance (*t* = 2.23, *p* = 0.069). A summary of the excitability measurements from the patient groups is provided in Table 2. With respect to controls, the refractoriness at 2 ms was larger in C9^+^ patients but not in C9^−^ patients (*t* = 3.76, *p* < 0.001; *t* = −1.23, *p* = 0.436). It is important to consider that these measurements are also dependent on temperature. For example, even with the dedicated warming procedure that was implemented to minimize temperature variability, a strong correlation between temperature and refractoriness at 2 ms was present in our data (*R* [95% CI] = −0.33 [−0.44, −0.20], *p* < 0.001). To ensure that the established effects attributed to C9orf72 carriership were not the result of minor variability in temperature, we performed a sensitivity analysis by adding temperature as a covariate to the models. Even with this addition, refractoriness at 2 ms, RRP, and S2‐accommodation remained significantly larger in C9^+^ patients than in C9^−^ patients (*t* = 4.37, *p* < 0.001; *t* = 3.19, *p* = 0.005; *t* = 2.59, *p* = 0.028). Apart from measurements that are associated with the refractoriness of the nerve, no other standard or composite excitability measurements were found to differ between the C9^+^ and C9^−^ ALS patients, including the SDTC and superexcitability that have diagnostic potential [13]. Between C9^−^ ALS patients and controls, we identified differences in the standard excitability measurements from the threshold electrotonus test, current–voltage test, and the recovery cycle (Table 2).

## 4 Discussion

Our study shows that carriers of the C9orf72 mutation, both asymptomatic individuals and patients, have unique profiles of peripheral nerve excitability that could be indicative of a unique electrophysiological phenotype. Based on one of the biophysically informed composite excitability measures, a reduction in Na^+^‐window currents was the most likely mechanism contributing to the difference between asymptomatic C9orf72 carriers and noncarriers, which could contribute to hypoexcitability. In patients, C9orf72 carriers most notably exhibited larger refractoriness during the recovery cycle compared to sporadic ALS patients. This increased refractoriness is likely dominated by slower recovery from inactivation of Na^+^‐channels that prolongs the influx of Na^+^‐ions and contributes to hyperexcitability. These findings highlight the utility of nerve excitability testing as a tool to study the asymptomatic phase in C9orf72 mutation carriers.

To date, no direct comparison of peripheral nerve excitability measurements had been made between asymptomatic C9orf72 mutation carriers and noncarriers from families with a history of ALS. One previous study compared asymptomatic carriers to healthy (population‐based) controls, but found no significant differences in the excitability of these two groups [21]. We identified a subtle difference in one of the biophysically informed composite excitability measurements (composite 3) when comparing the subset of asymptomatic carriers and noncarriers from the same families. Assuming that peripheral nerve excitability is influenced by multiple unknown genetic factors, the finding may be explained by the fact that accounting for kinships between the related individuals enhanced statistical power to detect otherwise unnoticeable differences in excitability between carriers and noncarriers, or carriers and healthy (population‐based) controls. Based on a previous extensive modeling study [40], a reduced value of composite 3 was indicative of smaller Na^+^‐window currents, which refer to the sustained inward currents that arise from the overlap between the activation‐ and inactivation curves of the Nav1.6 channels [43]. Although we cannot identify which specific membrane‐ and channel properties of Nav1.6 channels cause the observed excitability changes, the consequence of the smaller Na^+^‐window currents is that asymptomatic C9orf72 carriers are relatively hypoexcitable compared to noncarriers. Preclinical studies have provided supporting evidence that iPSC derived motor neurons from C9orf72 ALS patients undergo a brief period of hyperexcitability, followed by normalization and then hypoexcitability as they mature [19, 20, 44]. To provide further insights into the involved underlying mechanisms, future studies may consider expanding the standard excitability testing protocol with latent addition tests. These tests could help to elucidate which modality of nodal Na^+^ ion‐channels, transient or persistent, most likely contributes to the electrophysiological phenotype. Although this technique has been considered preferable to the strength‐duration test as it is less susceptible to passive membrane properties [45], at present its implementation in studies of ALS remains limited.

In patients with C9orf72 ALS, the refractory region of the recovery cycle was the most distinctive compared to sporadic ALS patients, as well as to healthy controls. Even though the refractoriness at 2 ms has a notable contribution to composite measurement 4, no differences were found in that measurement between the patient groups. This is partly because the other measures in this composite had a negligible contribution to the mean value of the composite measurement, indicating that the pattern of excitability changes in C9orf72 ALS patients with respect to sporadic patients was not adequately described by composite 4. Of note, in our previous modeling study, we were not able to identify the biophysical basis that may underlie the changes in this particular composite measurement [40]. In contrast, exclusive changes to the refractory period—as identified in the C9orf72 ALS patients—are known to be influenced by properties of the axon, the neuromuscular junction, or the muscle membrane [34, 43]. Changes to the excitability of the muscle membrane are less likely to be of relevance in ALS [46]. At the neuromuscular junction, conflicting results have been produced in mouse models of C9orf72 ALS [47]. If the site of abnormal refractoriness is restricted to the axon, it is generally accepted that the recovery from inactivation of Nav1.6 channels dominates the response [35], with a slower recovery from inactivation prolonging the influx of Na^+^‐ions into the axoplasm, which increases the refractoriness and causes hyperexcitability. Delineating the origin of altered refractoriness could help to unravel disease mechanisms in C9orf72 ALS. Supplementing the standard nerve excitability testing protocol with a recently developed double‐conditioned recovery cycle protocol could help to localize the origin of the excitability changes that were observed in our study [48]. Involvement of the neuromuscular junction could, alternatively, be explored in these patients by means of repetitive nerve stimulation [49]. We also identified larger S2‐accommodation of the depolarizing threshold electrotonus in C9orf72 ALS patients compared to sporadic patients. In the absence of other clear excitability changes, however, we were not able to reliably determine which membrane properties could have produced this abnormality.

Some limitations of our study merit consideration. Recordings of nerve excitability were obtained from only one arm at a single timepoint. However, patients and asymptomatic individuals with a medical history or concomitant presence of neuromuscular diseases in the examined arm were carefully excluded, thus minimizing other pathophysiological confounders beyond ALS or C9orf72 carriership. Some asymptomatic individuals were the sole participants from their families and could, therefore, not be compared to their direct relatives. Nevertheless, the majority of the study population consisted of related asymptomatic individuals, yielding the largest dataset of nerve excitability measurements in asymptomatic C9orf72 carriers to date. Only a small proportion of the ALS cohort was C9orf72 carrier (9%), which is consistent with the expected proportion in the general population [3]. This relatively small number of C9orf72 ALS patients, in combination with substantial variance in several excitability measurements, may inadvertently lead to reduced sensitivity to capture differences in these subgroups. Still, the fact that subtle changes in excitability could be detected in small numbers of C9orf72 ALS patients and asymptomatic carriers indicates that longitudinal assessment of these is warranted. These studies should emphasize recruitment strategies to expand the asymptomatic and patient C9orf72 cohorts in order to attain adequately powered results. Lastly, our study solely focused on the function and integrity of the peripheral motor neurons, and including other promising wet biomarkers (e.g., neurofilaments, creatine kinase or troponin T [50, 51]) could help to better characterize the study population. Ultimately, a combination of neuroimaging, neurophysiological, and wet biomarkers will be required in order to capture the full system dysfunction and to further advance our understanding of the pathogenesis of C9orf72 ALS.

ALS is hypothesized to be a multistep disorder, and sporadic ALS was found to require more steps to develop than genetic variants such as C9orf72 ALS [52]. Our results demonstrate that carriership of the C9orf72 mutation contributes to a unique electrophysiological phenotype. In asymptomatic carriers, the subtle excitability differences were most consistent with a hypoexcitable profile mediated by smaller Na^+^‐window currents. ALS patients with the C9orf72 mutation had larger refractoriness than sporadic ALS patients, which may be best explained by slower recovery from inactivation of Na^+^‐channels that promotes hyperexcitability due to prolonged influx of Na^+^‐ions. Given that nerve excitability changes occur before motor neuron loss can be objectively detected [13, 14, 15], tracking these measurements over time in asymptomatic C9orf72 carriers to identify a switch from hypoexcitability to hyperexcitability might help to reveal early signs of disease onset. Future studies applying these additional techniques longitudinally in C9orf72 carriers could, therefore, help further improve our understanding of the phenotype conversions of asymptomatic individuals into ALS.

## Author Contributions

All authors contributed to the conception and design of the study; D.J.L.S.G., H.S.G., B.T.H.M.S. contributed to the acquisition and analysis of data; all authors contributed to drafting the text or preparing the figures.

## Conflicts of Interest

The authors declare no conflicts of interest.

## Supporting information


**Table S1:** Summary of upper motor neuron findings from neurological examination in the asymptomatic C9orf72 carriers and noncarriers.

## Data Availability

The study data supporting the findings are available upon reasonable request via the corresponding author.
