# Supplementary material for: Nerve Excitability in Asymptomatic Carriers and Amyotrophic Lateral Sclerosis Patients With C9orf72
Source: Ann Clin Transl Neurol. 2025 Sep 9;12(12):2470–81. doi: 10.1002/acn3.70187 (PMC12698937; doi:10.1002/acn3.70187)
Supplement: Supplementary file 1 — Table S1: Summary of upper motor neuron findings from neurological examination in the asymptomatic C9orf72 carriers and noncarriers. [file ACN3-12-2470-s001.docx]

SUPPLEMENTARY MATERIAL

*Neurological examination of asymptomatic individuals*

Data from standardized neurological examinations were available for 45/47 asymptomatic individuals from families with a history of C9orf72 ALS. No lower motor neuron signs (e.g. fasciculations, weakness or muscle atrophy) were found in the population. Some instances of brisk or very brisk reflexes were identified in isolated limbs of participants from both the C9^+^ and C9^-^ groups, none of which were considered to be of pathophysiological relevance. A summary of the results of the upper motor neuron examination is presented in Supplementary Table 1.

**Supplementary Table 1**: summary of upper motor neuron findings from neurological examination in the asymptomatic C9orf72 carriers and noncarriers.

| **Characteristic** | **Asymptomatic C9^+^** | **Asymptomatic C9^-^** | ***P*** |
| --- | --- | --- | --- |
| Available in | 22 (96) | 23 (96) | - |
| Time since participation (months) | 0 (-0.6, 7.0) | 0.3 (0, 4.7) | 0.70 |
| **Bulbar region** | | |  |
| Dysarthria | 0 | 0 | 1 |
| Impaired tongue movements | 0 | 0 | 1 |
| Sustained glabella reflex | 2 (9) | 3 (13) | 0.98 |
| Masseter reflex | 0 | 0 | 1 |
| Snout reflex | 1 (5) | 2 (9) | 0.58 |
| Palmomental reflex (R/L) | 4 / 2 (19 / 10) | 1 / 3 (5 / 14) | 0.17 / 0.67 |
| **Cervical and thoracic region** | | |  |
| Elevated arm muscle tone (R/L) | 0 / 0 | 0 / 0 | 1^a^ |
| Biceps reflex (R/L) |  |  | 1^a^ |
| Normal | 16 / 16 (73 / 73) | 18 / 18 (78 / 78) |  |
| Brisk | 6 / 6 (27 / 27) | 5 / 5 (22 / 22) |  |
| Very brisk | 0 / 0 | 0 / 0 |  |
| Triceps reflex (R/L) |  |  | 0.94^a^ |
| Normal | 17 / 17 (77 / 77) | 20 / 20 (87 / 87) |  |
| Brisk | 5 / 5 (23 / 23) | 3 / 3 (13 / 13) |  |
| Very brisk | 0 / 0 | 0 / 0 |  |
| Deltoid reflex (R/L)^b^ | 5 / 7 (24 / 33) | 7 / 7 (33 / 33) | 0.50 / 1 |
| Trapezius reflex (R/L)^b^ | 2 / 2 (10 / 10) | 2 / 2 (10 / 10) | 1^a^ |
| Pectoral reflex (R/L)^b^ | 6 / 5 (29 / 24) | 3 / 3 (14 / 14) | 0.27 / 0.44 |
| Hofmann-Tromner reflex (R/L) | 0 / 1 (0 / 5) | 0 / 0 | 1^a^ |
| Abdominal reflex (R/L) | 22 / 22 (100 / 100) | 21 / 21 (91 / 91) | 1^a^ |
| **Lumbosacral region** | | |  |
| Elevated leg muscle tone (R/L) | 0 / 0 | 0 / 0 | 1^a^ |
| Knee jerk reflex (R/L) |  |  | 0.09 / 0.17 |
| Normal | 13 / 13 (59 / 59) | 19 / 18 (83 / 78) |  |
| Brisk | 9 / 9 (41 / 41) | 4 / 5 (17 /22) |  |
| Very brisk | 0 / 0 | 0 / 0 |  |
| Ankle jerk reflex (R/L) |  |  | 0.74 / 0.95 |
| Normal | 16 / 15 (73 / 68) | 19 / 19 (83 / 83) |  |
| Brisk | 6 / 6 (27 / 27) | 4 / 4 (17 / 17) |  |
| Very brisk | 0 / 1 (0 / 5) | 0 / 0 |  |

Data are presented as median (IQR), evocable/graded reflex count (%) or count per side (% per side) when performed on both sides (R/L); no percentage is shown in case of a zero count. C9^+^, C9^-^ = positive or negative for the C9orf72 repeat expansion.

^a^Equal proportion and *P*-values for both sides

^b^Missing in one C9^+^ and two C9^-^
